# Case report: Molecular characterization of adult atypical teratoid rhabdoid tumor and review of the literature

**DOI:** 10.3389/fonc.2025.1510439

**Published:** 2025-02-20

**Authors:** Miguel A. Hernandez-Rovira, Michelle Connor, Robert C. Osorio, Emilie Russler-Germain, Robert Schmidt, Gabrielle W. Johnson, Julie Silverstein, Sonika Dahiya, Nyssa Fox Farrell, Mia C. Weiss, Gregory J. Zipfel, Jiayi Huang, Dimitrios Mathios

**Affiliations:** ^1^ School of Medicine, Washington University in St. Louis, St. Louis, MO, ;United States; ^2^ Department of Neurosurgery, Washington University School of Medicine, St. Louis, MO, ;United States; ^3^ School of Medicine, University of California, San Francisco, San Francisco, CA, ;United States; ^4^ Department of Pathology and Immunology, Washington University in St. Louis, St. Louis, MO, ;United States; ^5^ Division of Endocrinology, Metabolism and Lipid Research, Department of Medicine, Washington University in St. Louis, St. Louis, MO, ;United States; ^6^ Department of Otolaryngology, Washington University in St. Louis, St. Louis, MO, ;United States; ^7^ Division of Oncology, Department of Medicine, Washington University in St. Louis, St. Louis, MO, ;United States; ^8^ Brain Tumor Center, Siteman Cancer Center, Washington University School of Medicine, St. Louis, MO, ;United States; ^9^ Department of Radiation Oncology, Washington University in St. Louis, St. Louis, MO, ;United States

**Keywords:** atypical teratoid rhabdoid tumor, hyperprogression, case report, adult, systematic review

## Abstract

**Background and importance:**

Atypical teratoid rhabdoid tumors (ATRTs) are typically aggressive pediatric tumors with a median survival of 11 months. Due to the paucity of cases in adults, the clinical behavior of these pathologies is not well understood. Here we present the case of a 41-year-old female patient with postoperative hyperprogression of a sellar ATRT and provide a detailed description of the molecular composition of this tumor, the protocol used to treat this patient, and the ultimate outcome of this patient.

**Clinical presentation:**

The patient is a 41-year-old woman who presented with headaches and double vision. MRI revealed a sellar/suprasellar mass with involvement of bilateral cavernous sinuses. Following the quick symptom progression, resection of the tumor with exploration of the bilateral cavernous sinuses was performed, with a final pathologic diagnosis of ATRT-MYC, a known subtype of ATRT. The tumor recurred within 1 month of surgery, attaining a size equivalent to its preoperative state. Postoperatively, the patient received craniospinal radiation and adjuvant chemotherapy with an excellent response but had a recurrence of the tumor in the brainstem 1 year after her diagnosis and died 13 months after presentation.

**Discussion:**

Sellar ATRT in adults is an exceedingly rare entity. The detailed description of our case highlights the aggressiveness of these tumors and the utility of postoperative chemotherapy and radiation, but also the inevitable progression of these tumors along the craniospinal axis.

**Conclusion:**

Sellar ATRTs should be considered in the differential diagnosis of a sellar/suprasellar mass, especially in women in their 40s. Emphasis should be placed on accurate diagnosis and quick postoperative recovery with early initiation of adjuvant radiation and chemotherapy.

## Introduction

Atypical teratoid rhabdoid tumors (ATRTs) are a rare and aggressive central nervous system cancer that carries a poor prognosis (1–3). First described as a unique entity in 1996 (1), ATRTs almost exclusively present in the pediatric population (3). However, these tumors can also rarely affect adults, with just over 100 documented cases reported in the literature (4). Of those, 47 are in the sellar/suprasellar region. Prior studies show that these tumors develop primarily in female patients and the supratentorial region (4).

While this disease has been comprehensively studied in pediatric patients, the clinical course of ATRTs is incompletely understood in adults. Here we present a case of postoperative hyperprogression of sellar ATRT in a 41-year-old otherwise healthy female patient. We further analyzed all published cases of ATRTs to further characterize patient response to treatment.

## Case presentation

**Figure 1:**
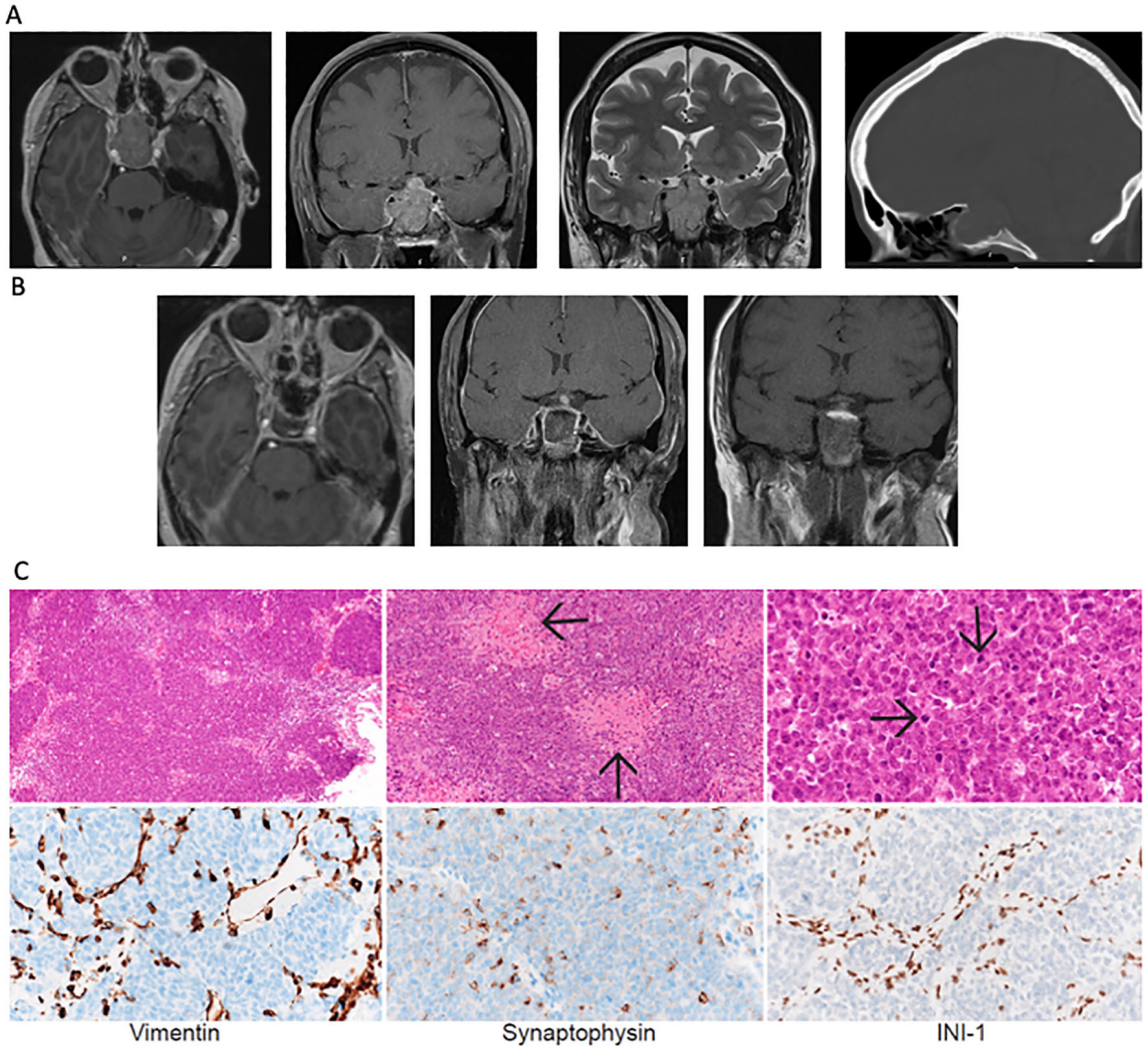
**(A)** Axial and coronal FLAIR and T2 coronal MRI sequences and sagittal head CT imaging showing tumor in the sellar region at presentation with encasement of the internal carotid artery (Knosp grade 4). **(B)** Postoperative MRI following expanded endonasal resection revealed residual tumor near the right internal carotid artery. **(C)** Low- (10x), medium- (20x) and high- (60x) hematoxylin and eosin-stained sections show a densely-cellular, solid/lobular high-grade tumor with abundant necrosis. Immunohistochemical stains for Vimentin and Synaptophysin show scattered positivity of tumor cells. Immunohistochemical stain for INI-1 shows loss of protein expression in tumor, with retained expression in non-neoplastic interstitial vascular and stromal cells.

A 41-year-old female patient presented to the emergency department with a 6-week history of intermittent headaches. A computed tomography (CT) scan of the head revealed a 3.1-cm sellar mass with suprasellar extension. The follow-up magnetic resonance imaging (MRI) found an enhancing sellar mass consistent with a pituitary adenoma. Serum electrolytes were notable for Na^+^ at 127 mmol/L (normal range: 135–145 mmol/L). Endocrine workup was consistent with secondary adrenal insufficiency with low ACTH (3.6 pg/mL) and cortisol (0.3 mcg/dL), secondary hypothyroidism with low free T4 (0.43 ng/dL), inappropriately normal TSH (TSH 1.03 mU/L) and hyperprolactinemia (80.6 ng/mL, normal <25 ng/mL). Levothyroxine at 100 mcg daily and hydrocortisone at 40 mg twice daily were prescribed, with clinical follow-up 3 days later for evaluation of resection, after which new symptoms of intractable headache, diplopia, and difficulty in opening her right eye developed and progressed over 4 days. Repeat head CT and MRI revealed right cavernous carotid artery encasement by the lesion and mass effect on the optic chiasm (**Figure 1A**). Intravenous (IV) hydrocortisone at 50 mg every 8 h was started, and surgery was scheduled.

Expanded endoscopic endonasal resection was performed. Intraoperatively, a soft, hemorrhagic tumor filling the entirety of the sphenoid sinus was identified. Given the preoperative hormonal deficits, the aggressive appearance of the lesion intraoperatively, and the tumor adhesion to the pituitary gland, the entire pituitary gland was removed. Exploration of the bilateral cavernous sinuses revealed no invasion on the left but significant invasion/adherence on the right, resulting in an incomplete resection. Given the suprasellar extension of the tumor, a high-flow cerebrospinal fluid (CSF) leak was encountered and was closed with an inlay and onlay synthetic dural graft, followed by a vascularized pedicled nasoseptal flap. Postoperatively, the patient’s third nerve palsy and electrolyte abnormalities resolved. Imaging studies showed a residual tumor lateral to the right internal carotid artery (**Figure 1B**).

**Figure 2:**
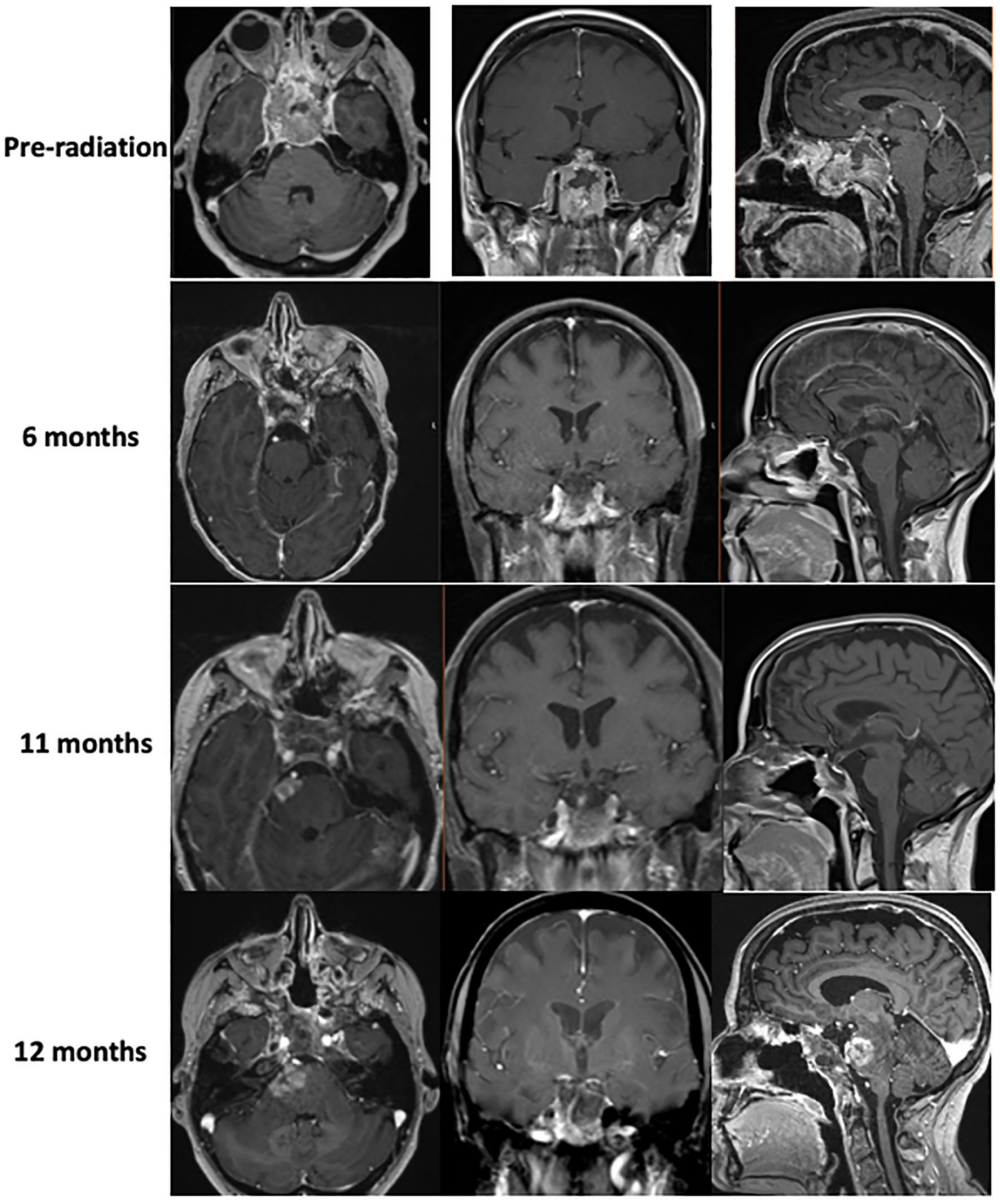
MR imaging showing tumor hyperprogression prior to radiation therapy, as well as tumor response to chemotherapy 6 and 11 months out from diagnosis. Imaging shows evidence of brainstem invasion at 12 months.

The pathology examination revealed a solid proliferation of poorly differentiated malignant cells with rhabdoid appearance and large areas of necrosis (**Figure 2**) with extensive invasion of the surrounding sinonasal mucosa and adenohypophysis. Immunohistochemical staining revealed focal synaptophysin and rare chromogranin-A positivity within the tumor. The tumor cells were notable for diffuse loss of INI1 and were negative for CAM5.2, SOX10, CCD3, and CD20 (**Figure 1C**). The final pathologic diagnosis was an atypical teratoid rhabdoid tumor. Next-generation sequencing revealed two SMARCB1 mutations (M217fs*12, splice site 94-1G>C), with 0 mutations/Mb tumor mutational burden. This tumor was categorized by methylome analysis as ATRT, MYC-subtype, WHO grade 4. Spinal MRI and CSF cytology were negative for leptomeningeal spread.

The patient later presented with a pulmonary embolism and right lower extremity deep vein thromboses (DVTs), which were treated with apixaban. At follow-up, the patient again reported ptosis of the right eye, double vision, and numbness in the V2 distribution (**Figure 2**), prompting early (4 weeks after surgery) proton craniospinal irradiation with 36 Gy and a 54 Gy boost to the original tumor site over 6 weeks. The patient was continued on levothyroxine and hydrocortisone, but estrogen replacement was deferred due to recent DVTs. Radiation was followed by adjuvant chemotherapy with cisplatin, cyclophosphamide, and vincristine based on the pediatric ACNS0333 protocol (2), which was adapted for her age. While undergoing chemotherapy, she developed neutropenic fever twice, which required hospitalization and treatment with antibiotics and blood transfusions, prompting a reduction in the dose of Cytoxan and replacement of cisplatin with carboplatin.

**Figure 3:**
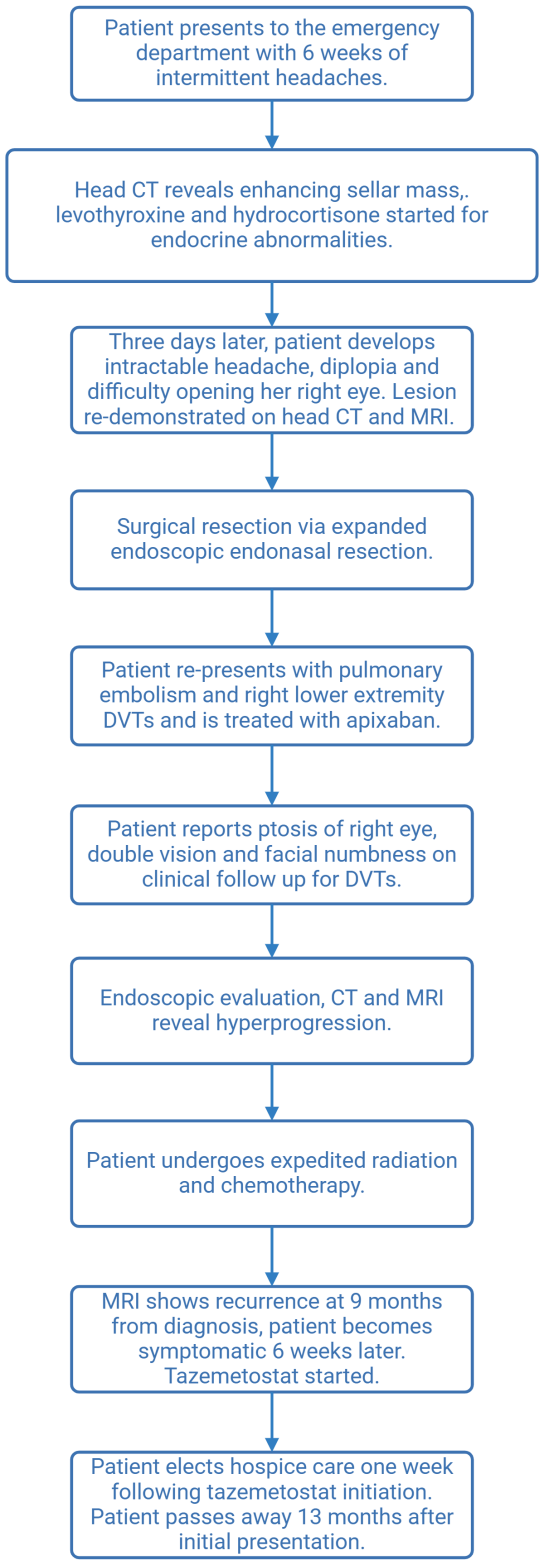
Flowchart describing patient course.

Brain MRI 9 months after the diagnosis revealed enhancement in the pons adjacent to the high-dose radiation field. Approximately 6 weeks later, the patient presented again to the hospital with difficulty walking and a left facial droop, with the repeat brain MRI result showing an increased lesion size in the right pons with surrounding edema extending to the bilateral middle cerebellar peduncles and midbrain, which was concerning for tumor recurrence (**Figure 2**). The area of recurrence was just outside of the 54-Gy isodose line of the high-dose radiation field and had received at least 40–50 Gy. Tazemetostat (an *EZH2* inhibitor) was initiated to target the SMARCB1 deletion in the tumor. However, the patient and her family elected hospice care within 1 week of initiation. The patient died 13 months after the initial presentation. See **Figure 3** for a representation of the patient’s clinical course.

## Discussion

ATRTs are rare WHO grade 4 CNS tumors that predominantly affect children and are defined by a characteristic alteration of either *INI1* or *BRG1*, resulting in the loss of nuclear expression (1–3, 5). These tumors are currently grouped into three subtypes based on clinical and molecular characteristics: ATRT-SHH, ATRT-TYR, and ATRT-MYC. ATRT-SHH presents at a median age of 20 months as an infratentorial and/or supratentorial tumor with overexpression of sonic hedgehog (SHH) and Notch pathway components. This subtype is associated with a higher frequency of heterozygous *SMARCB1* mutations and a lower frequency of loss or deletion of this gene than the other two groups. ATRT-TYR typically presents at a median age of 12 months as an infratentorial tumor with band-like contrast enhancement on MRI. Molecular analysis reveals an overexpression of tyrosinase (TYR) and other components of the melanosomal pathway and the bone morphogenetic pathways. These tumors often present with complete or partial chromosome 22 deletion and increased chromatin accessibility. Finally, ATRT-MYC presents as a supratentorial tumor at a median age of 27 months, displaying an overexpression of the *MYC* oncogene along with s the *HOXC* gene cluster, with homozygous inactivation of *SMARCB1*. The MRI imaging typically shows strong peritumoral edema (6, 7).

**Figure 4:**
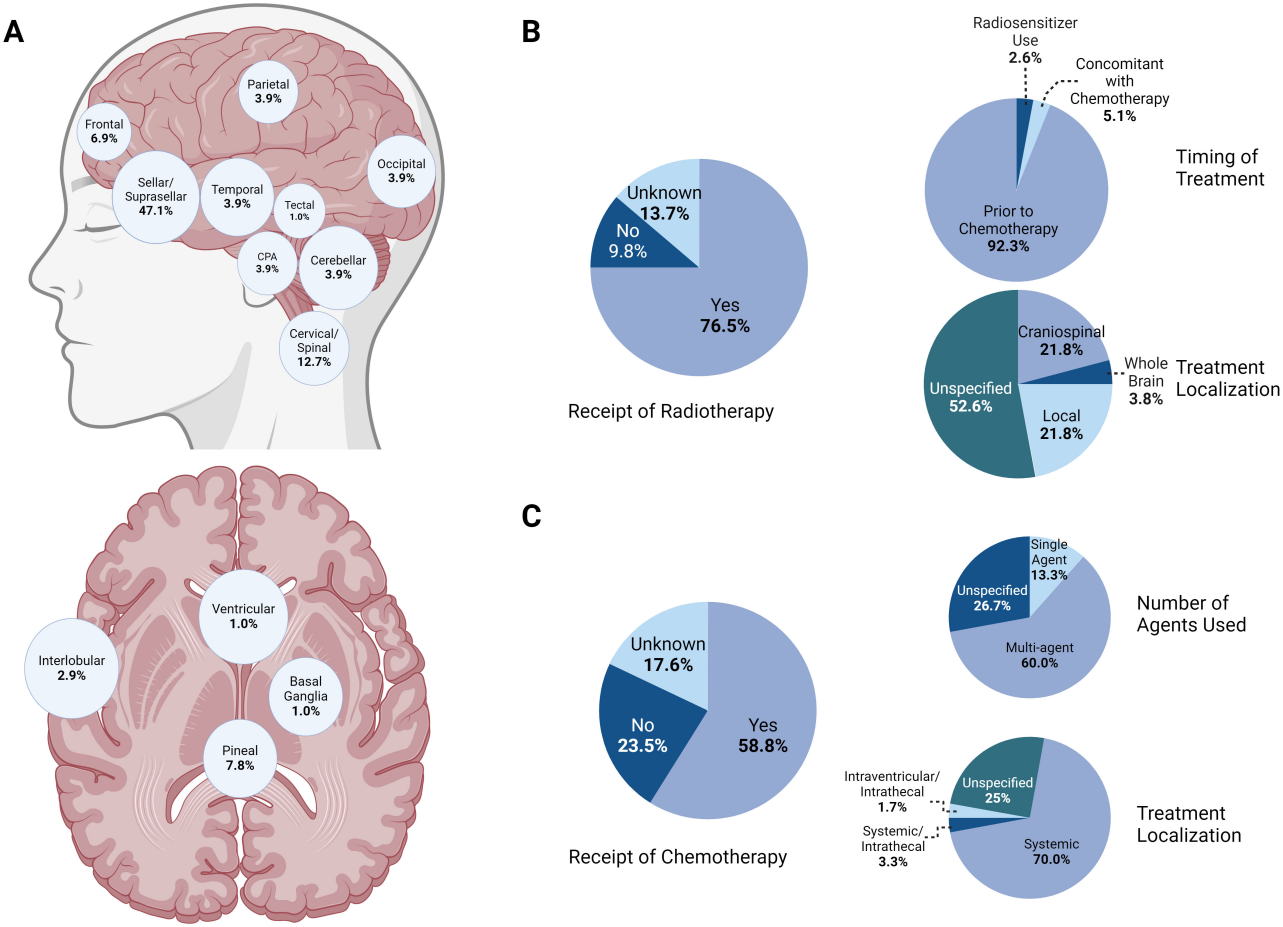
**(A)** Anatomic distribution of known ATRT cases demonstrating a majority of cases in the sellar/suprasellar region, followed by the cervical/spinal and pineal regions. **(B)** Summary of trends in radiotherapy for known ATRT patients. A majority (76.5%) of patients received adjuvant radiotherapy, with most of those undergoing treatment prior to receiving chemotherapy. While the specific regimen was not described for most patients, localized and craniospinal radiation were employed equally, with a minority of cases featuring whole-brain treatment. **(C)**Summary of trends in chemotherapy for known ATRT patients. Most patients (58.8%) received adjuvant chemotherapy, mostly following radiotherapy and with systemic agents.

Currently, there are 47 adult cases of sellar ATRT reported in the literature, yet full molecular descriptions are lacking. Likewise, while the management of pediatric ATRTs has been extensively discussed in the literature, there is no consensus on the therapeutic approach to adult ATRTs (**Figure 4**). In this report, we have not only provided a detailed molecular profile of this tumor in our patient but also thoroughly described the therapeutic approach and clinical management of this disease. After disease progression at 10.5 months post-diagnosis, the patient began treatment with the *EZH2* inhibitor tazemetostat. This medication inhibits the function of the catalytic subunit of polycomb repressive complex 2 (PRC2), which leads to chromatin compaction and gene repression via histone 3 lysine 27 trimethylation. PRC2 function is regulated by a functional antagonism with BRG1/BRM-associated factors (BAGs), a family of complexes whose canonical form contains the SMARC family of proteins, including SMARCB1 (9). SMARCB1 downregulation or loss of function is associated with decreased transcription, leading to malignancy. EZH2 inhibition corrects the imbalance caused by SMARCB1 inhibition, decreasing the inhibitory effects of PRC2. Tazemetostat is traditionally used in the treatment of epithelioid sarcoma with INI1/SMARCB1 loss of function (10), which also characterizes ATRTs (11). Preclinical studies have explored additional pharmacological treatments, with one showing synergy between carboplatin and 6-diazo-5-oxo-L-norleucine to extend survival in orthotopic models of ATRT-MYC (8).

The biological mechanisms for the female predominance of ATRT are still poorly understood. Notably, the *SMARCB1* gene is located on chromosome 22q11, like the neurofibromatosis 2 (*NF2*) gene, which is associated with meningiomas, also a female-predominant pathology. While no mechanism for these epidemiological differences has been uncovered, one study identified no differences in the expression of progesterone, estrogen, and androgen receptors between male and female meningioma samples obtained from surgical resection. Thus, it is unlikely that incidence differences are due to hormonal effects (12). While no such studies have been conducted in the context of ATRT, the absence of hormonal contributions to meningioma incidence may reflect intrinsic cellular mechanisms that may similarly apply to ATRT.

Given the dearth of treatment protocols for ATRTs in the adult population, we aimed to review the current state of the literature on sellar ATRTs to identify patterns in approaches leading to longer survival times. Our patient’s presentation corroborated the most common characteristics seen in adult ATRT (**Supplementary Table 1**), which presents mostly in female patients with an average age of 46.5 ± 13.8 years. Like our patient, 26 patients in the literature underwent endoscopic endonasal resection, resulting in subtotal resection of a sellar mass. Our patient underwent proton craniospinal radiotherapy, which was performed in an additional case (13). One of the sellar ATRT patients (14) had a clinical course similar to our patient’s, with tumor progression occurring within 2 weeks of initial surgery. This patient received a comprehensive chemotherapy regimen of vincristine, doxorubicin, cyclophosphamide, ifosfamide, carboplatin, and etoposide, followed by craniospinal radiation. This contrasts with the clinical course of our patient, who received radiation prior to chemotherapy with a more conservative regimen of cisplatin, cyclophosphamide, and vincristine. While our patient achieved clinical stability for close to 1 year, the other patient passed away within 2 months.

Although the majority of our patient's clinical course approximated that of other patients, its early postoperative course was marked by hyperprogression. Tumor hyperprogression, also called a tumor flare, is described as a disease with rapid progression exceeding that expected without treatment. This phenomenon is most often observed following immunotherapy, particularly with anti-PD-1/PD-L1 agents (15). The main characterization of tumor hyperprogression following operative management has been studied in the context of pancreatic ductal adenocarcinoma, and the authors found an association between hyperprogression and decreased survival (Zou et al.) (16). This phenomenon suggests that achieving a negative margin resection with unacceptable morbidity to the patient is unlikely to lead to improved survival and is likely to be associated with more surgical complications, which may delay adjuvant treatment that appears to be effective in eliminating this disease. Therefore, emphasis should be placed on accurate tissue diagnosis and safe tumor debulking with minimally invasive surgical approaches that can accelerate the patient’s ability to move toward adjuvant treatment initiation and help relieve the patient’s symptoms. It is worth noting that our patient did not exhibit a local recurrence, but rather a distant recurrence in the pons, which had received a lower dose of radiation at the time of CSI.

To determine the role of chemotherapy and radiation therapy in the management of adult ATRT, we analyzed the relationship between each treatment protocol and overall survival (OS) across all patients (**Supplementary Table 1**). Spearman’s *r* showed a significant negative correlation between age and OS (-0.2705, *p = 0*.0113), with patients younger than 40 years old having a greater OS than those older than 40 years (22.0 months vs. 9 months, *p = 0*.0039). Patients who did not undergo post-surgical radiation (*n* = 10) or chemotherapy (*n* = 22) survived for an average of 1.9 and 18.0 months, respectively, whereas those who underwent the full course of treatment survived for an average of 26.9 months. This difference was shown to be statistically significant via Mann–Whitney tests (*p* = <0.0001 and *p* = <0.0001, respectively). However, it is difficult to discern whether patient survival was impacted negatively by their treatment regimen or by greater deterioration at baseline, as low post-surgical performance may have prevented patients from undergoing further treatment. While adjuvant chemotherapy increased the OS, no differences were observed between single-agent and multi-agent chemotherapy (*p* = *0*.6161, Mann–Whitney test) or between systemic and localized chemotherapy (*p* = *0*.6595, Mann–Whitney test). Moreover, no differences between localized, whole-brain, and craniospinal radiation (*p* = *0*.9425, Kruskal–Wallis test) or between traditional and proton radiation (*p* = *0*.8594, Mann–Whitney test) were identified. Cisplatin radiosensitization or simultaneous chemoradiation were not associated with changes in survival (*p* = *0*.5130, Kruskal–Wallis test). Tumor location within the sellar/suprasellar region did not significantly impact the OS (*p = 0*.1438). Furthermore, the extent of resection and the use of minimally invasive techniques did not impact the outcomes (*p = 0*.7610 and *p = 0*.2408, respectively).

## Limitations

The studies included in our literature search are reports of individual cases, which limit the nature of the conclusions that can be drawn from the statistical analysis performed due to discrepancies in the level of detail between the reports. Moreover, the sample sizes for each treatment protocol were limited, decreasing the statistical power of the analyses comparing radiation and chemotherapy regimens. Upon careful analysis of the relevant studies, we identified that the most common reason for the absence of data was a vague description of the radio- or chemotherapeutic regimen or no description of the patient’s clinical course. This was most common in pathological studies of this entity. A number of patients received treatment at institutions different from those where surgery was performed, which limited the description of the patient’s course. One patient had planned adjuvant radio- and chemotherapy, without any details provided. Finally, a number of patients were reported in abstracts or other media that lacked a full description of the clinical course.

## Conclusion

We have reported a case of tumor hyperprogression in a sellar ATRT in an adult and provided detailed molecular analyses of the tumor and the long-term outcome of the patient. Our case emphasizes the notion that these tumors are not surgically curable. Therefore, emphasis should be placed on accurate diagnosis, quick surgical recovery, relief of symptoms related to compression of vital structures, and initiation of adjuvant treatment that involves a combination chemotherapy regimen, and craniospinal radiation with a boost to the primary tumor site.

## Data Availability

The raw data supporting the conclusions of this article will be made available by the authors, without undue reservation.
