# Supplementary material for: Case report: Molecular characterization of adult atypical teratoid rhabdoid tumor and review of the literature
Source: Front Oncol. 2025 Feb 20;15:1510439. doi: 10.3389/fonc.2025.1510439 (PMC11882417; doi:10.3389/fonc.2025.1510439)
Supplement: Supplementary file 1 [file Table1.docx]

**Table 1: Summary of demographic and clinical characteristics of ATRT patients.**

| Patient | Age | Gender | Location | Surgery | Radiation Regimen | Chemotherapy Regimen | Overall Survival | Survival | Recurrence |
| --- | --- | --- | --- | --- | --- | --- | --- | --- | --- |
| 1 | 34 | F | Parietal | N/A | N/A | N/A | 6 | Yes | N/A |
| 2 | 21 | F | Spinal Cord | N/A | N/A | N/A | 6 | Yes | N/A |
| 3 | 71 | M | Pineal | Biopsy via endoscopic approach | Cranial, follow up spinal | Etoposide, carboplatin | 8 | Yes | N/A |
| 4 | 20 | F | Sellar | Yes | Yes | Chemotherapy (unspecified) | 28 | Yes | Yes, local |
| 5 | 31 | F | Sellar | N/A | Yes | N/A | 9 | No | N/A |
| 6 | 45 | M | Cerebellar | Yes | Cranial | Chemotherapy (unspecified) | 15 | Yes | No |
| 7 | 43 | F | Spinal Cord | Cervical, Incomplete Lumbar, | Cervical Spine, 50 Gy photon beam radiotherapy in 2 Gy daily fractions Lumbar Spine, 50 Gy Posterior fossa, 46 Gy Thoracic Spine, 37.5 Gy at 2.5 Gy per fraction Thoracolumbar 12 Gy boost | Temozolomide, later docetaxel | 30 | No | Metastasis to the lumbar spine Cervical recurrence |
| 8 | 23 | M | Right frontal suprasylvian | Incomplete resection via craniotomy followed by repeat craniotomy | Radiotherapy (unspecified) | N/A | 1 | No | Yes, intracranial |
| 9 | 23 | M | Posterior left frontal | Resection via craniotomy | Radiotherapy (unspecified) | Chemotherapy (unspecified) | 30 | Yes | N/A |
| 10 | 25 | F | Frontal | Gross total resection via craniotomy | Gamma Knife for recurrence 2 years later Adjuvant radiotherapy for recurrences | Chemotherapy for recurrence | 204 | Yes | Yes, intracranial, 6 total |
| 11 | 42 | M | Right frontoparietal | Incomplete resection | Radiotherapy (unspecified) | Chemotherapy (unspecified) | 18 | Yes | No |
| 12 | 56 | F | Sellar | Incomplete resection via transsphenoidal approach | Stereotactic irradiation (34 Gy to the center, 17 Gy to the periphery and) Extended local brain, 50.4 Gy Spinal, 43.2 Gy, for recurrence | None | 23 | No | Yes, disseminated across the brainstem, lumbar spinal cord and cauda equina Second dissemination to the chiasma, ventricles, brain stem surface, and spinal cord |
| 13 | 18 | M | Right frontotemporal | Right craniotomy | Radiotherapy (unspecified) | None | 4 | No | No |
| 14 | 19 | M | Supratentorial | Biopsy only | Craniospinal radiation | Combination therapy with vincristine, dactinomycin and cyclophosphamide; cispla- tin, doxorubicin (DOX), and imidazole carboximide | 25 | No | N/A |
| 15 | 33 | F | Pineal | Subtotal resection | Craniospinal radiation | Adjuvant chemotherapy | 13 | Yes | Yes |
| 16 | 46 | F | Sellar | N/A | N/A | N/A | N/A | N/A | N/A |
| 17 | 22 | F | Right occipital | Initial gross total resection followed by second surgery | Extended focal conventional radiation therapy, 60 Gy | Ranimustine 100 mg/m2 upon initial radiotherapy and three million internal units of interferon-beta for 7 days | 5 | No | Yes, local, after 3 months Dissemination one month after second operation |
| 18 | 26 | M | Cervical | Subtotal resection at 6 months of age Second surgery for recurrence | Whole-brain radiotherapy, 34.2 Gy over 4 weeks Posterior fossa radiotherapy, 5.4 Gy over one week | None | 5 | No | Yes, at 26 years of age |
| 19 | 33 | F | Pineal | Subtotal resection | Craniospinal radiation | Ifosphamide, Etoposide and Carboplatin (ICE) protocol in 3-week cycles DOX, vincristine and temozolomide for second recurrence | 18 | Yes | Yes, regrowth to original size at 3 weeks Second recurrence after eight cycles of ICE |
| 20 | 61 | F | Sellar | Incomplete resection followed by second resection | N/A | None | N/A | N/A | Yes, postoperatively |
| 21 | 57 | F | Sellar | Resection via transsphenoidal approach | Radiotherapy (unspecified) | DOX and cisplatin, three cycles | 6 | Yes | Yes, one month postoperatively |
| 22 | 19 | F | Supratentorial | Near total resection | Craniospinal, 23.4 Gy with tumor boost to 55.8 Gy | Vincristine, cisplatin, cyclophosphamide with peripheral blood stem cell transplant | 18 | Yes | No |
| 23 | 20 | F | Pineal | Biopsy only | Gamma Knife, 18 Gy | None | 27 | No | Yes, after 12 months |
| 24 | 37 | M | Left temporal | Surgical resection twice (for original tumor and recurrence) | Proton beam radiotherapy | Cytoxan/vincristine and ICE, four alternating cycles | 43 | Yes | Yes, 5 months postoperatively |
| 25 | 24 | F | Right temporal with suprasellar extension | Gross total resection via temporal craniotomy | Craniospinal radiation, 36 Gy with tumor boost to 54 Gy | ICE, six cycles, dosing per agent:  ifosfamide: 1800 mg/m2 for four days etoposide: 100 mg/m2 for four days carboplatin: AUC 5 for two days | 11 | Yes | No |
| 26 | 58 | F | Right parietotemporal | Stereotactic craniotomy | Radiotherapy, 60 Gy in 30 fractions | None | 20 | No | Yes, local and leptomeningeal |
| 27 | 60 | F | Sellar | Near total resection via transphenoidal approach | Stereotactic radiotherapy, 34 Gy to center and 17 Gy to the periphery over 3 months Second round of stereotactic radiotherapy was performed following metastasis | DOX and vinorelbine, nine cycles Carboplatin and paclitaxel every 21 days | 30 | No | Yes, metastasis to the lung |
| 28 | 43 | F | Sellar | Resection via transsphenoidal surgery | Radiotherapy (unspecified) | None | N/A | N/A | N/A |
| 29 | 22 | M | Left occipital | None | Involved-field radiation, 54 Gy | Vincristine, etoposide, cisplatin and cyclophosphamide, four cycles | 15 | No | No |
| 30 | 44 | F | Right occipital | Gross total resection via craniotomy | Cranial radiation | ICE | 9 | Yes | No |
| 31 | 44 | F | Sellar, suprasellar | Partial resection via translabial transphenoidal approach Second partial resection via basal interhemispheric approach | Radiotherapy (unspecified) | ICE, five cycles in three-week intervals | 17 | No | Yes, dissemination to the right cerebellar hemisphere and vermis, spinal cord Metastasis to the lung |
| 32 | 42 | F | Sellar, suprasellar | Subtotal resection with transphenoidal approach | Craniospinal radiation and spinal irradiation with proton beam therapy, 36 Gy with 18 Gy local boost, cisplatin radiosensitizer | 11 cycles of vincristine, cisplatin, adriamycin, etoposide or cyclophosphamide alternating weekly with vincristine, etoposide, ifosfamide and carboplatin for 10 months | 24 | Yes | No |
| 33 | 65 | M | Spinal Cord | T11–L1 laminectomy and subtotal tumor resection T4-5 laminectory with tumor resection for recurrence | External beam radiotherapy, 1.67 gy/fraction, 5 fractions per week for 30 fractions total Adjuvant radiotherapy following first recurrence | None | 24 | No | Yes, at T4, then to sacrum/pelvis |
| 34 | 22 | F | Cerebellopontine angle | Gross total resection | Radiotherapy (unspecified) | Chemotherapy (unspecified) | 15 | No | No |
| 35 | 48 | F | Sellar | Transphenoidal resection (gross total resection) Repeat resection for recurrence | Pituitary and craniospinal radiation following chemotherapy | Vincristine, DOX and cyclophosphamide alternated with ICE, six cycles | 2 | No | Yes, after 2 weeks postoperatively, and 10 days after recurrence |
| 36 | 36 | F | Sellar | Transphenoidal resection (incomplete) Second transphenoidal resection followed by craniotomy for debulking Three additional surgeries for debulking | Stereotactic radiotherapy External beam radiation | Temozolomide Vincristine, cyclophosphamide, and adriamycin-D (VCA) with cisplatin and VP16 for recurrences | 30 | No | Yes, multiple recurrences and growth into the left temporal lobe |
| 37 | 45 | F | Sellar | Gross total resection via transsphenoidal approach | Stereotactic radiotherapy | None | 6 | No | Yes, increase in cranial nerve deficits |
| 38 | 19 | F | Spinal Cord | Resection through L2 and L3-L4 laminectomies | Lumbar spine irradiation, 1.8 Gy/day for a total of 54Gy | Alternating DOX, ICE and VCA followed by consolidation with carboplatinum and thioepa Peripheral blood stem cell transplant Vinorelbine, cyclophosphamide and celecoxib, antiangiogenesis for recurrence | 36 | Yes | Yes, local extension from T12 to L5 |
| 39 | 30 | M | Cerebellopontine angle | Intratumoral debulking | Radiotherapy (unspecified) | Chemotherapy (unspecified) | N/A | N/A | No |
| 40 | 24 | M | Left frontal | Total resection | Craniospinal radiation, 36 Gy with focal radiotherapy to 18 Gy | Cyclophosphamide, cisplatin and vincristine Peripheral blood stem cell transplantation | 30 | Yes | No |
| 41 | 24 | M | Left occipital | Partial resection | Whole brain radiotherapy, 60 Gy | Temozolomide and bevacizumab, postoperative | 60 | Yes | No |
| 42 | 22 | F | Sagittal frontal | Partial resection | None | None | 0.13 | No | No |
| 43 | 69 | F | Sellar | Transphenoidal resection | Extended focal radiotherapy | Temozolomide Maintenance temozolomide, five days in 28-day cycles, 200mg/body | 24 | Yes | No |
| 44 | 43 | F | Sellar | Subtotal resection via transsphenoidal approach | None | None | 1 | No | Yes, twenty days postoperatively |
| 45 | 51 | M | Tectal | Suboccipital craniectomy for biopsy Infratentorial supracerebellar resection with gross total resection | Fractionated proton beam radiotherapy, 54 Gy at 1.8 Gy/fractions over 6 weeks | Temozolomide, 75 mg/m2 daily, then 150 mg/m2 for five days, followed by five cycles of 200 mg/m2, concurrent with radiotherapy | 12 | Yes | No |
| 46 | 29 | F | Left cerebellopontine | Left retrosigmoid craniotomy | Intensity-modulated radiotherapy, 54 Gy in 30 fractions Conformal radiotherapy to C5-T6 vertebral levels, 30 Gy in 10 fractions Conformal radiotherapy to T12-L4 vertebral levels, 30 Gy in 10 fractions | ICE, two 14-day cycles Intrathecal liposomal cytarabine, 50 mg biweekly, six doses | 5 | No | Yes, leptomeningeal metastases to the cervical and thoracic cord |
| 47 | 35 | M | Suprasellar | Pterional craniotomy | Craniospinal radiation, 36 Gy in 20 fractions, with 20Gy local boost in 12 fractions Lumbar spine, 8 Gy in 4 fractions | VCA, six cycles Peripheral blood stem cell transplantation | 30 | Yes | Yes, leptomeningeal metastasis |
| 48 | 23 | F | Left temporal | Subtotal resection via craniotomy | None | None | 8 | No | No |
| 49 | 35 | M | Right frontal suprasylvian | Gross total resection Partial temporal lobe resection for progression 12 years after diagnosis Stereotactic biopsy 6 months after second surgery | Radiotherapy, 40 Gy, for recurrence | None | 168 | Yes | Yes, recurrence 4 years after diagnosis Progression into right temporal lobe 10 years after diagnosis Massive growth of right temporal mass following resection |
| 50 | 19 | M | Pineal | Stereotactic biopsy followed by parieto-occipital combined transcortical and interhemispheric appproach resulting in near-total resection | Radiotherapy, 54 Gy in 1.8 Gy/fraction 5 times weekly) | Intrathecal chemotherapy, single dose DOX, one cycle ICE, three cycles Cyclophosphamide and vincristine, five alternating cycles | 18 | Yes | No |
| 51 | 36 | F | Sellar | Subtotal resection via transphenoidal approach | Radiotherapy, 60Gy in 30 fractions | Vincristine, concurrent with radiotherapy ICE, six cycles | 74 | Yes | No |
| 52 | 26 | F | Sellar | Surgical resection | Radiotherapy concomitant with chemotherapy | Multiagent chemotherapy incuding cisplatin and etoposide | 33 | No | No |
| 53 | 21 | F | Sellar | Surgical resection | Radiotherapy concomitant with chemotherapy | Multiagent chemotherapy incuding cisplatin and etoposide | 35 | No | No |
| 54 | 43 | F | Spinal Cord | C1-3 decompressive laminectomy resulting in GTR | Radiotherapy, 50.4 Gy in 28 fractions over 7 weeks | None | 6 | Yes | No |
| 55 | 22 | F | Left perirolandic | Surgical resection (GTR) | N/A | N/A | N/A | N/A | N/A |
| 56 | 62 | F | Sellar | Craniotomy | Radiotherapy (unspecified) | None | N/A | N/A | N/A |
| 57 | 42 | F | Sellar | Subtotal resection via transphenoidal approach twice | Stereotactic radiosurgery (performed twice), 16 Gy and 14 Gy Conventional radiotherapy, 60 Gy, for recurrence | Temozolomide, 150 or 200 mg/m2 daily for five days in 28-day cycles Paclitaxel, 80 mg/m2 daily on days 1, 8, 15 in 28-day cycles, for recurrence | 11 | No | Yes, 6 months after diagnosis |
| 58 | 31 | F | Sellar | Subtotal surgical resection | None | None | 2 | No | No |
| 59 | 36 | F | Sellar | Subtotal surgical resection | Radiotherapy (unspecified) | Chemotherapy (unspecified) | 22 | Yes | No |
| 60 | 47 | F | Sellar | Subtotal surgical resection | Radiotherapy, 20 Gy in 10 fractions | Chemotherapy (three agents, unspecified) | 62 | Yes | No |
| 61 | 65 | F | Sellar | Subtotal surgical resection | Radiotherapy, 54 Gy in 30 fractions, cisplatin sensitizer | Vincristine, cisplatin, DOX and cyclophosphamide | 23 | No | Yes, leptomeningeal and spinal metastases |
| 62 | 20 | F | Sellar | Surgical resection | N/A | Initial chemotherapy unknown ICE with peripheral blood cell transplant, for recurrence | 120 | No | Yes, four years postoperatively |
| 63 | 46 | F | Sellar | Surgical resection | N/A | N/A | 0 | No | No |
| 64 | 48 | F | Sellar | N/A | N/A | N/A | 4 | Yes | No |
| 65 | 66 | M | Sellar | N/A | N/A | N/A | 54 | Yes | No |
| 66 | 59 | F | Sellar | Subtotal resection via transphenoidal endoscopic resection | Radiotherapy (unspecified) | None | 2 | No | Yes |
| 67 | 27 | M | Right basal ganglia, internal capsule and thalamus | Subtotal resection via right frontal craniotomy for interhemispheric transcallosal internal decompression | None | None | 0 | No | No |
| 68 | 51 | M | Sellar | Biopsy via expanded endoscopic endonasal approach | Focal photon radiation therapy, 30.6 Gy at 1.8 Gy/fraction, daily Craniospinal radiation, 23.4 Gy at 1.8 Gy/fraction, daily | Temozolomide, 75 mg/m2, concurrent with radiation | 9 | Yes | No |
| 69 | 33 | F | Sellar | Biopsy via endoscopic, transsphenoidal approach Transsphenoidal decompression | Craniospinal radiation, 36 Gy in 20 fractions, with 18 Gy local boost | Alternating ICE-DOX-VCA | 36 | Yes | No |
| 70 | 55 | F | Sellar | Initial transsphenoidal resection Right frontal craniotomy for progression | None | None | 1.5 | Yes | Yes, six weeks after diagnosis |
| 71 | 62 | F | Sellar | Resection via transcranial right subfrontal approach | None | None | 2 | No | No |
| 72 | 27 | F | Sellar | Surgical transsphenoidal debulking | N/A | N/A | N/A | N/A | N/A |
| 73 | 19 | F | Spinal Cord | Laminectomy at L4, L5, S1 and S2 levels | Radiation therapy, 54 Gy in 30 fractions | N/A | N/A | N/A | Yes, local recurrence two months after final radiotherapy session Spread to lower thoracic spinal cord up to T6 and new lesion at T1 |
| 74 | 24 | M | Left parietal | Gross total resection via left parietal craniotomy | Three-dimensional local radiotherapy, 54 Gy in 1.8 Gy fractions | DOX, cyclophosphamide, vincristine, ifosfamide, cisplatin, etoposide, and methotrexate, three nine-week courses, Intrathecal augmentation | 48 | Yes | No |
| 75 | 23 | M | Left frontal dural | N/A | Radiotherapy (unspecified) | Temozolomide for 3 months | 72 | Yes | No |
| 76 | 55 | M | Parietal | N/A | N/A | N/A | N/A | N/A | N/A |
| 77 | 30 | F | Ventricular | Total resection via suboccipital craniotomy | N/A | N/A | N/A | N/A | N/A |
| 78 | 29 | M | Pineal | Near total resection via infratentorial supracerebellar approach Resection of residual via occipital interhemispheric transcallosal resection | Whole brain radiation, 54 Gy in 30 fractions over 6 weeks | Vincristine, methotrexate with leucovorin rescue, cyclophosphamide, etoposide and cisplatin, 2 cycles | 22 | Yes | No |
| 79 | 41 | F | Pineal | Gross total resection via infratentorial supracerebellar approach | Radiotherapy (unspecified) | Alternating DOX-ICE-VCA | 31 | Yes | No |
| 80 | 71 | M | Pineal | Biopsy via endoscopic third ventriculostomy | Cranial radiation,46 Gy in 23 fractions over 5 weeks Palliative spinal radiation from C7 to T4 (20 Gy in 5 fractions) | Temozolomide,150 mg/m2 on days 1-5 of a 28-day cycle | 8 | No | Yes, spinal |
| 81 | 70 | F | Sellar | Biopsy/resection via right frontotemporal craniotomy | Fractionated external beam cranial radiation, 30 Gy in 10 fractions | Etoposide and topotecan, intraventricular/intrathecal | 6 | No | No |
| 82 | 40 | F | Sellar | Subtotal resection via transnasal endoscopic approach | Radiotherapy (unspecified) | Chemotherapy (unspecified) | 1 | No | N/A |
| 83 | 55 | F | Sellar | Surgical resection | Radiotherapy (unspecified) | Vincristine | N/A | No | N/A |
| 84 | 43 | F | Sellar | Resection via transsphenoidal surgery | Cranial radiation, 59.5 Gy in 25 fractions | ICE, six cycles | 48 | Yes | N/A |
| 85 | 58 | F | Sellar | Resection via left frontal midline craniotomy, transcallosal approach Extended resection of residual via extended transnasal craniotomy | Radiotherapy (unspecified) | Chemotherapy (unspecified) | N/A | N/A | No |
| 86 | 29 | F | Sellar | Subtotal resection via endoscopic transnasal sphenoidal approach | Radiotherapy, 60 Gy in 30 fractions Local Gamma Knife radiotherapy | None | 8 | No | No |
| 87 | 43 | F | Sellar | Subtotal resection via endoscopic transnasal sphenoidal approach | Radiotherapy, 52 Gy in 28 fractions | Cisplatin and dacarbazine, six cycles | 4 | No | No |
| 88 | 50 | F | Sellar | Gross total resection via endoscopic transnasal sphenoidal approach | None | None | 1 | No | No |
| 89 | 52 | F | Sellar | Subtotal resection via endoscopic transnasal sphenoidal approach | None | None | 2 | No | No |
| 90 | 80 | F | Sellar | Subtotal resection via endoscopic transnasal sphenoidal approach | None | None | 1 | No | No |
| 91 | 25 | M | Right trigeminal | Subtotal resection following progression via combined suboccipital retrosigmoid and temporal approaches | Cyberknife surgery, 18 Gy in 3 fractions over 3 days | None | 3.5 | No | Yes, within 2 months of diagnosis |
| 92 | 38 | F | Cerebellar | Gross total resection via suboccipital craniotomy and C1 laminectomy with telovelar tonsillar approach | Craniospinal radiation, 36 Gy in 20 fractions, with 19.8 Gy boost in 11 fractions | Cisplatin, etoposide, and cyclophosphamide, six cycles | 12 | Yes | No |
| 93 | 19 | F | Spinal Cord | Subtotal resection | Radiotherapy (unspecified) | Chemotherapy (unspecified) | 40 | No | N/A |
| 94 | 20 | F | Cerebellar | N/A | Radiotherapy (unspecified) | Chemotherapy (unspecified) | 84 | Yes | No |
| 95 | 27 | M | Cerebellar | N/A | N/A | N/A | N/A | N/A | N/A |
| 96 | 29 | F | Cerebellopontine angle | Subtotal resection | N/A | N/A | 18 | Yes | N/A |
| 97 | 44 | M | Spinal Cord | N/A | Radiotherapy (unspecified) | DOX, vincristine and cyclophosphamide, six cycles | N/A | N/A | N/A |
| 98 | 43 | F | Sellar | Subtotal resection | Radiotherapy (unspecified) | ICE, three cycles VCA, three cycles Temozolomide, 12 cycles Thiotepa, one cycle Thiotepa-carboplatin, one cycle Autologous peripheral blood cell transplant | 6 | Yes | N/A |
| 99 | 36 | F | Left parietal | Gross total resection via left parietal craniotomy | Radiation therapy, 54 Gy in 2 Gy fractions over 27 days | None | 103 | Yes | No |
| 100 | 21 | M | Left temporal base | Subtotal resection | Cranial radiation, 60 Gy | Chemotherapy (multifasceted) | 72 | No | Yes, after four years and six years |
| 101 | 60 | F | Sellar | Resection via endoscopic endonasal approach | Radiotherapy, 54 Gy in 30 fractions | Cyclophosphamide, DOX, vincristine and etoposide | N/A | N/A | Yes, after 3 weeks |
| 102 | 41 | F | Sellar | Subtotal resection via expanded endoscopic endonasal approach | Craniospinal radiation, 36 Gy with proton boost to 54 Gy for 6 weeks | Cisplatin, cyclophosphamide and vincristine | 13 | No | Yes, 4 weeks postoperatively |
